# *SQSTM1* mutation: Description of the first Tunisian case and literature review

**DOI:** 10.1002/mgg3.1543

**Published:** 2020-11-02

**Authors:** M. Akkari, I. Kraoua, H. Klaa, H. Benrhouma, T. Ben Younes, A. Rouissi, M. Chaabouni, I. Ben Youssef‐Turki

**Affiliations:** ^1^ LR18SP04, Department of Child and Adolescent Neurology University of Tunis El Manar National Institute Mongi Ben Hmida of Neurology Tunis Tunisia; ^2^ Laboratory of Medical Analyzes and Human Genetics Jasmins Medical Center Tunis Tunisia

**Keywords:** cognitive decline, degenerative ataxia, dystonia, ophthalmoparesis, *SQSTM1* mutation

## Abstract

**Background:**

Mutations in *SQSTM1* gene have been recently identified as a rare cause of progressive childhood neurodegenerative disorder. So far, only 25 patients from 10 unrelated families were reported.

**Methods and results:**

We report on the first Tunisian case of an 11‐year‐old girl with cerebellar ataxia, chorea and ophthalmoparesis. Brain MRI was normal. Whole‐exome sequencing revealed a homozygous mutation c.823_824del(p.Ser275Phefs*17) in *SQSTM1* gene (GenBank: NM_003900.4).

**Conclusion:**

By pooling our data to the data of literature, we delineated the phenotypic spectrum and stressed on genetic heterogeneity of this rare neurodegenerative disease.

## Graphical abstract

Mutations in *SQSTM1* gene have been recently identified as a rare cause of progressive childhood neurodegenerative disorder. We report on the first Tunisian case of an 11‐year‐old girl with cerebellar ataxia, chorea and ophthalmoparesis.

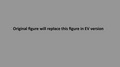

## 1 INTRODUCTION

Heterozygous *SQSTM1* variants have been associated with amyotrophic lateral sclerosis, Paget's disease, frontotemporal dementia, and distal hereditary myopathy with rimmed vacuoles (Le Ber et al., 2013). Recently, recessive mutations in *SQSTM1* causative of progressive childhood‐onset neurodegenerative disorder characterized by cognitive decline, ataxia, dystonia, and gaze palsy were identified (Haack et al., 2016; Majcher et al., 2015; Muto et al., 2018; Vedartham et al., 2019; Zúñiga‐Ramírez et al., 2019). We report on the first Tunisian patient with homozygous mutation of *SQSTM1*.

## 2 OBSERVATION

An 11‐year‐old girl was referred to our department for evaluation of movement disorders. She was born to unrelated healthy parents. The pregnancy was complicated by maternal CMV infection in the 4th month. Serologic tests of CMV were repeatedly negative in the amniotic fluid and a fetal infection was ruled out. Our patient had a normal psychomotor development: she was able to walk independently at the age of 13 months. At the age of 9 years, the teacher remarked writing difficulties. Visual problem was suspected, however, ophthalmologic examination was normal. Subsequently, she developed deterioration in school performance, involuntary movement disorders, gait instability as well as balance and coordination problems.

Clinical examination revealed cerebellar signs with dysarthria, ataxic gait without enlargement of the support polygon, a postural instability, hypotonia, Dysdiadochokinesia, and dysmetria in finger‐to‐nose test. She also had generalized chorea. The study of oculomotricity revealed a downgaze palsy with a restriction of abduction. She had no telangiectasias and no pyramidal signs. A standardized intelligence quotient (IQ) assessment revealed a full scale IQ of 90 but working memory difficulties. Brain and spine MRI were normal. Laboratory tests, including hemogram, creatine kinase, IgA, and thyroid function tests were unremarkable. Electromyography and muscle biopsy were normal.

A written informed consent was obtained from the parents and whole‐exome sequencing was performed using a SureSelect Human All Exon 38 Mb enrichment kit. Co‐segregation analysis revealed homozygous frameshift variant c.823_824del(p.Ser275Phefs*17) in SQSTM1 (GenBank: NM_003900.4) as the likely candidate and confirmed that the mutation was inherited from heterozygous carrier parents. No other mutations were detected. The analysis of the gene dosage using exome depth did not indicate any copy‐number variation. Our patient has been treated with coenzyme Q10 (100 mg daily), L‐carnitine (1 g daily), vitamin E (200 mg twice daily), vitamin C (500 mg per day), and Piracetam (600 mg twice daily). She also underwent occupational and speech therapies.

## 3 DISCUSSION

We report on the first Tunisian child with cerebellar ataxia, chorea, and ophthalmoparesis due to recessive mutation in *SQSTM1*. Sequestosome 1 (*SQSTM1*), encoding for p62 protein, is an adaptor protein involved in a variety of key cellular processes including oxidative stress response, apoptosis, and cell differentiation (Le Ber et al., 2013; Seibenhener et al., 2007). In addition, it plays a critical role in the degradation of ubiquitinated substrates, by its function as a selective autophagy receptor (Katsuragi et al., 2015). Therefore, mutations of *SQSTM1* are closely linked to neurodegenerative diseases through the autophagy failure (Zhou et al., 2013).

This progressive childhood neurodegenerative disease caused by *SQSTM1* mutations is rare. Indeed, so far, only 25 patients related to 10 families have been reported (Haack et al., 2016; Muto et al., 2018; Vedartham et al., 2019; Zúñiga‐Ramírez et al., 2019). Demographic, clinical, imaging, and genetic findings of our patient and those of the 25 published cases are summarized in Table 1.

**TABLE 1:** Demographic and genetic data of index patient and reported patients with *SQSTM1* mutations (Haack et al., 2016; Majcher et al., 2015; Muto et al., 2018; Vedartham et al., 2019; Zúñiga‐Ramírez et al., 2019)

	P1	P2	P3	P4	P5	P6	P7	P8	P9	P10	P11	P12	P13
	F1	F1	F2	F2	F3	F3	F4	F4	F4	F4	F4	F4	F4
Sex	M	M	M	M	F	M	M	F	M	M	F	M	M
Origin	Jordan	Jordan	Italy	Italy	Iran	Iran	Iran	Iran	Iran	Iran	Iran	Iran	Iran
Consanguinity	+	+	+	+	+	+	+	+	+	+	+	+	+
Age of onset	9	14	6	12	10–12	10–12	10	9	10	10	10	11	10
Age at last examination	35	24	41	35	33	29	30	29	28	16	26	17	39
Ataxia (100%)	+	+	+	+	+	+	+	+	+	+	+	+	+
Dysarthria (100%)	+	+	+	+	+	+	+	+	+	+	+	+	+
Cognitive decline (96%)	+	+	+	+	+	+	+	+	+	+	+	+	+
Gaze palsy (88.4%)	–	+	+	+	+	+	+	+	+	+	+	+	+
Dystonia (61.5%)	+	+	+	+	+	+	–	–	–	–	–	–	–
Dyskinesia (15.3%)	–	–	+	+	+	+	–	–	–	–	–	–	–
Chorea (23%)	+	–	+	+	+	+	–	–	–	–	–	–	–
Hypergonadotropic hypogonadism (7.7%)	–	–	+	+	–	–	–	–	–	–	–	–	–
MRI	ND	N	CA	ND	ND	ND	ND	ND	ND	ND	ND	ND	ND
SQSTM1 Variants	c.823_824del	c.823_824del	c.301 + 2 T > A	c.301 + 2 T > A	c.934_936delinsTGA	c.934_936delinsTGA	c.875_876insT	c.875_876insT	c.875_876insT	c.875_876insT	c.875_876insT	c.875_876insT	c.875_876insT
Course	stable	Stable	Stable	stable	Wheel chair	Wheel chair	ND	ND	ND	ND	ND	ND	ND

	P14	P15	P16	P17	P18	P19	P20	P21	P22	P23	P24	P25	P26 (our patient)
	F5	F5	F6	F6	F6	F7	F7	F7	F8	F9	F9	F10	F11
Sex	M	M	M	F	F	F	F	F	F	F	M	F	F
Origin	Mexico	Mexico	Germany	Germany	Germany	Emirates	Emirates	Emirates	Finland	Kirdish	Kirdish	India	Tunisia
Consanguinity	–	–	–	–	–	ND	ND	ND	+	+	+	+	–
Age of onset	7	7	10	12	15	10	10	10	7	8	8	8	9
Age at last examination	27	24	45	42	33	31	18	12	18	33	17	11	11
Ataxia	+	+	+	+	+	+	+	+	+	+	+	+	+
Dysarthria	+	+	+	+	+	+	+	+	+	+	+	+	+
Cognitive decline	+	+	–	+	+	+	+	+	+	+	+	+	+
Gaze palsy	+	+	+	+	–	+	+	+	–	+	+	+	+
Dystonia	+	+	+	+	+	+	+	+	–	–	+	–	+
Dyskinesia	–	–	–	–	–	–	–	–	–	–	–	–	–
Chorea	–	–	–	–	–	–	–	–	–	–	–	–	+
Iridoplegia (3.8%)	+	–	–	–	–	–	–	–	–	–	–	–	–
Mydriatic pupils (7.7%)	+	+	–	–	–	–	–	–	–	–	–	–	–
Hearing loss (11.5%)	–	–	–	–	–	+	+	+	–	–	–	–	–
MRI	CA	N	ND	IA	IA	CA	CA	CA	CA	ND	ND	ND	ND
SQSTM1 variants	c.257_259delins35 and c.301+1G > T	c.257_259delins35 and c.301+1G > T	c.2 T > A	c.2 T > A	c.2 T > A	c.311_312del	c.311_312del	c.311_312del	c.286C>T	c.286C>T	c.286C>T	c.712_713insTCCTCCGAGTGTGAATTTCCTGA	c.823_824del
Course	Stable	Stable	Wheel chair	Wheel chair	Wheel chair	ND	ND	ND	ND	ND	ND	ND	Stable

*SQSTM1* gene (GenBank: NM_003900.4).

Abbreviations: −, absent; +, present; CA, cerebellar atrophy; F, female; IA, iron accumulation; M, male; N, normal; ND, not determined.

By pooling our case with those of the literature, the phenotypic spectrum seems very large. This neurodegenerative disease, caused by biallelic SQSTM1 mutations, is panethnic. In fact, reported patients were from Europe, Mexico, India, and the Middle East (Haack et al., 2016; Muto et al., 2018; Vedartham et al., 2019; Zúñiga‐Ramírez et al., 2019). To date, this is the first case reported in Africa. Consanguinity was found in seven families. The mean age of onset was 9 years (extreme 6–15 years). Sex ratio was 1.27.

Cerebellar Ataxia was found in all patients regardless of origin. Ophthalmoparesis and cognitive impairment were the commonest presenting symptoms (22 patients). Dystonia and chorea were noted in 15 and five cases, respectively. Dysautonomic features such excessive sweating and orthostatic hypotension with additional features like iridoplegia and anisocoria were reported by Zuniga‐Ramirez et al (two patients) (Zúñiga‐Ramírez et al., 2019). Three patients presented a hearing loss (Haack et al., 2016), and two presented a hypergonadotropic hypogonadism (Muto et al., 2018).

These findings were not observed in our patient. Brain MRI revealed a cerebellar atrophy in six patients and signal abnormalities in basal ganglia with iron accumulation in two individuals (Haack et al., 2016).

The mean age at last examination was 27.7 years. In these studies, the course of this disease is characterized by a relatively slow progression, and only a fifth of reported patients lost their ability to walk between the age of 16 and 32 years.

To date, 10 different mutations affecting the ubiquitin‐associated domain of *SQSTM1* are currently known, of which four are deletions (c.311_312del, c.934_936delinsTGA, c.257_259delins35, and c.823_824del), two are insertions (c.875_876insT and c.712_713insTCCTCCGAGTGTGAATTTCCTGA), four are substitutions (c.286C>T, c.2 T > A, c.301 + 2 T > A, and c.301+1G > T) (Haack et al., 2016; Muto et al., 2018; Vedartham et al., 2019; Zúñiga‐Ramírez et al., 2019). In our study, we found the same mutation (c.823_824del) as reported in two Jordan siblings and the same clinical phenotype (Zúñiga‐Ramírez et al., 2019). However, data show that the same mutation in SQSTM1 array can cause different phenotypic expressions. In fact, clinical symptoms can vary greatly even among affected members of the same family carrying the same mutation (Haack et al., 2016; Muto et al., 2018; Zúñiga‐Ramírez et al., 2019). Thus, there is controversy with regard to the genotype–phenotype correlation (Leach et al., 2006).

Recently, heterozygous SQSTM1 mutations have also been found in other diseases like Paget's disease (PDB), amyotrophic lateral sclerosis (ALS), and frontotemporal dementia (FTLD). Furthermore, causal relationships have been found between mutations of SQSTM1 and the occurrence of these diseases (Le Ber et al., 2013): Recent studies have confirmed the presence of p62‐positive inclusions in spinal motor neurons and frontal cortex, in SQSTM1 mutation carriers (Teyssou et al., 2013).

In contrast to PDB‐associated SQSTM1 mutations predominantly affecting the Ubiquitin‐Associated domain, the coding mutations in ALS/FTLD patients are widespread, affecting the regions essential for p62's functions such as the promoter regions (Rubino et al., 2012). Further studies are necessary to better investigate the role of p62 in the pathogenesis of these diseases.

## 4 CONCLUSION

SQSTM1 mutation is a rare cause of neurodegenerative disease characterized by progressive ataxia movement disorders and gaze palsy. Through our study, we highlight the importance of whole‐exome sequencing in the diagnosis of rare neurodegenerative disorders. Description of further cases, will allow to better understand the disease and to develop therapeutic trials.

## CONFLICT OF INTEREST

The authors declare no conflict of interest.

## AUTHOR'S CONTRIBUTIONS

All authors contributed equally to this work.
